# Human plasma enhances the expression of Staphylococcal microbial surface components recognizing adhesive matrix molecules promoting biofilm formation and increases antimicrobial tolerance *In Vitro*

**DOI:** 10.1186/1756-0500-7-457

**Published:** 2014-07-17

**Authors:** Anthony P Cardile, Carlos J Sanchez, Meghan E Samberg, Desiree R Romano, Sharanda K Hardy, Joseph C Wenke, Clinton K Murray, Kevin S Akers

**Affiliations:** 1 Department of Medicine, Infectious Disease Service, Brooke Army Medical Center, 3551 Roger Brooke Drive, JBSA Fort Sam Houston, TX 78234, USA; 2 Extremity Trauma and Regenerative Medicine Task Area, United States Army Institute of Surgical Research, JBSA Fort Sam Houston, TX, USA

**Keywords:** Human plasma, Biofilm, *Staphylococcus aureus*, Microbial surface components recognizing adhesive matrix molecules (MSCRAMMs), Vancomycin

## Abstract

**Background:**

Microbial biofilms have been associated with the development of chronic human infections and represent a clinical challenge given their increased antimicrobial tolerance. *Staphylococcus aureus* is a major human pathogen causing a diverse range of diseases, of which biofilms are often involved. Staphylococcal attachment and the formation of biofilms have been shown to be facilitated by host factors that accumulate on surfaces. To better understand how host factors enhance staphylococcal biofilm formation, we evaluated the effect of whole human plasma on biofilm formation in clinical isolates of *S. aureus* and the expression of seven microbial surface components recognizing adhesive matrix molecules (MSCRAMMs) known to be involved in biofilm formation by quantitative real-time PCR. We also evaluated whether plasma augmented changes in *S. aureus* biofilm morphology and antimicrobial resistance.

**Results:**

Exposure of clinical isolates of *S. aureus* to human plasma (10%) within media, and to a lesser extent when coated onto plates, significantly enhanced biofilm formation in all of the clinical isolates tested. Compared to biofilms grown under non-supplemented conditions, plasma-augmented biofilms displayed significant changes in both the biofilm phenotype and cell morphology as determined by confocal scanning laser microscopy (CLSM) and scanning electron microscopy (SEM), respectively. Exposure of bacteria to plasma resulted in a significant fold-increase in MSCRAMM expression in both a time and isolate-dependent manner. Additionally, plasma-augmented biofilms displayed an increased tolerance to vancomycin compared to biofilms grown in non-supplemented media.

**Conclusions:**

Collectively, these studies support previous findings demonstrating a role for host factors in biofilm formation and provide further insight into how plasma, a preferred growth medium for staphylococcal biofilm formation enhances as well as augments other intrinsic properties of *S. aureus* biofilms. Consequently, these findings indicate that incorporation of host factors may be necessary to better replicate *in vivo* conditions and for the best utility of a clinical biofilm assay to evaluate the process of biofilm formation and treatments.

## Background

*Staphylococcus aureus* is a significant human pathogen that causes a wide range of infections. The ability of *S. aureus* to colonize and establish biofilms, a surface-attached microbial community surrounded by a self-produced polymeric matrix, is a central pathogenic event contributing to disease in humans [1]. Biofilms are implicated as a significant factor contributing to chronic human infections [2-4], and represent a major challenge to modern medicine given their recalcitrance to antimicrobials and host mechanisms of clearance. Biofilm formation is a complex process involving distinct phases of attachment, accumulation, and maturation. The attachment of staphylococci and subsequently the accumulation phases of biofilm development are predominantly mediated by different types of bacterial adhesins. More specifically, a class of surface proteins known as the microbial surface components recognizing adhesive matrix molecules (MSCRAMMs), which in addition to virulence, are responsible for mediating initial attachment to both naïve tissues and various biomaterials [5-7].

Data continues to accumulate regarding *S. aureus* biofilm formation, but there is increasing evidence that *in vitro* biofilm assays may not accurately represent *in vivo* biofilms [8]. Factors potentially causing discrepancies between *in vivo* and *in vitro* conditions include the presence of host proteins, of which human plasma is the best characterized [5-9]. Plasma is a major component of blood [normally approximating 55%, volume/volume (v/v)] and is composed of coagulation factors, albumin, globulins and other factors [9-11]. Most body fluids consist of plasma filtrates and proteins present in plasma are also found at varying concentrations in human body fluids, to include (percent, v/v): burn wound exudates (10-44%), acute soft tissue wound exudates (23-36%), interstitial fluid (10-27%), nasal secretions (15-45%), ascitic fluid (4-26%), lymphatic fluid (10-50%), and synovial fluid (1-73%) [12-17]. The importance of host proteins in facilitating biofilm formation is highlighted by studies demonstrating that medical implants are often coated by various host matrix proteins, serving to enhance bacterial attachment and biofilm formation *in vivo*[7,8] as well as *in vitro* where the use of plasma has been shown to promote biofilm growth [18,19].

To better understand how host factors, in particular those within human plasma, augment biofilm formation in *S. aureus*, herein we examined the effect of human plasma on biofilm formation in clinical isolates, and evaluated its effects on the expression of staphylococcal MSCRAMMs important to biofilm formation. Furthermore, we also evaluated the effects of plasma on biofilm/bacterial morphology and changes in resistance to antimicrobials.

## Results and discussion

### Human plasma enhances biofilm formation by clinical isolates of *S. aureus*, in part through increased MSCRAMM expression

**Figure 1:**
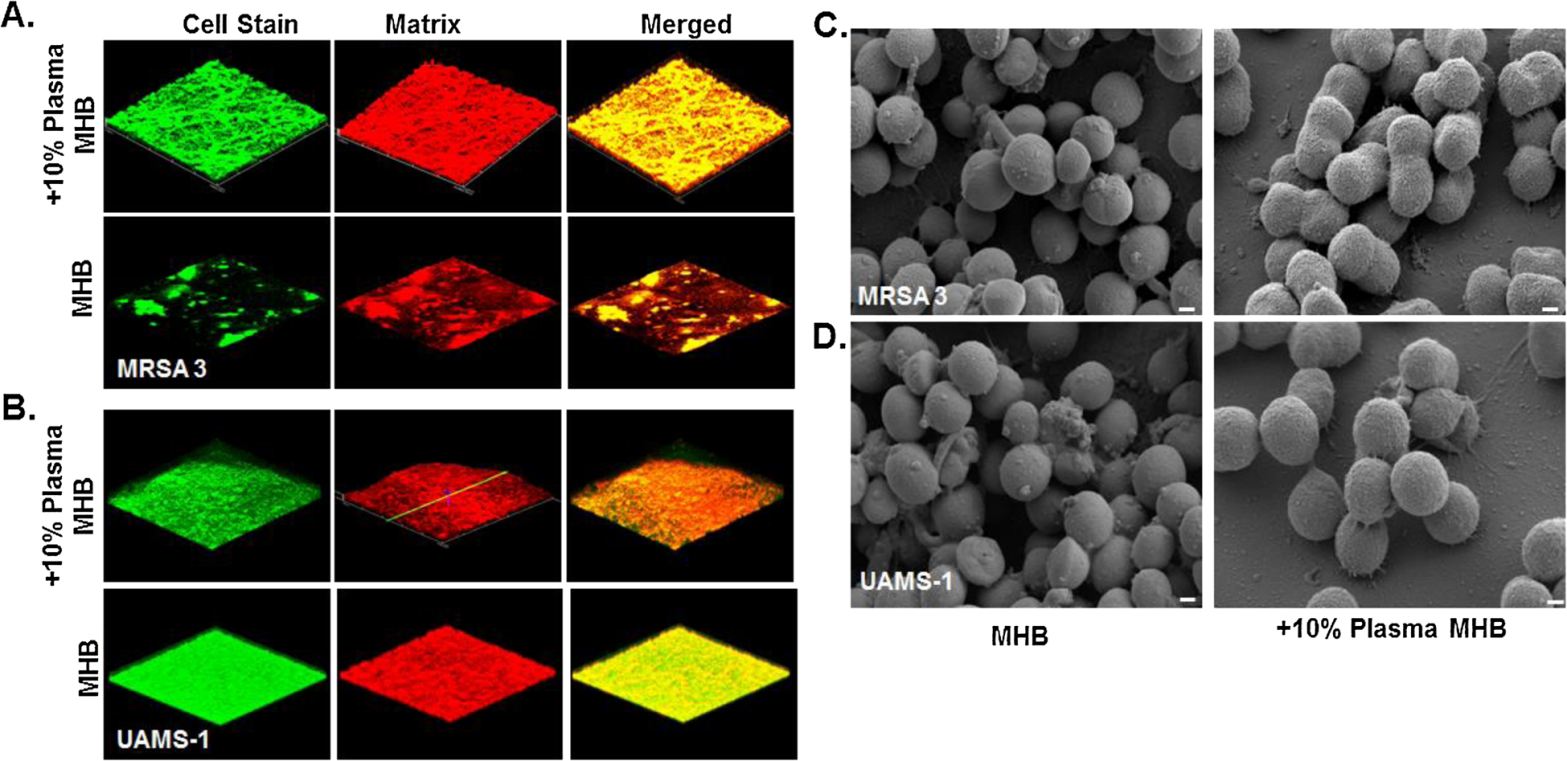
**Visualization of staphylococcal biofilms grown in the presence of human plasma by CLSM and SEM. A-B)** CLSM images biofilms of a methicillin-resistant (MRSA-3) and methicillin susceptible (UAMS-1) isolates of *S. aureus* grown overnight on coverslips in media supplemented with or without human plasma (10% Plasma). Biofilms were stained with a bacterial and a biofilm matrix stain to visualize the bacterial cells and extracellular polymeric matrix, respectively. Images were captured at 20X magnification. **C-D)** SEM analysis of biofilms of the clinical isolates listed above, following exposure to human plasma. Images were captured at 40,000X. Inlayed size bars represent 200 nm.

Previous studies have shown that supplementing media, as well as coating surfaces, with human plasma can facilitate *S. aureus* attachment and promote biofilm accumulation *in vitro*[18,19]. Consistent with these studies, supplementation of media with plasma, and to a much lesser extent when used to coat plates, was observed to enhance biofilm formation for all of the clinical isolates tested herein (Table 1). Interestingly, analysis of staphylococcal biofilms by CLSM, also demonstrated that the biofilm phenotype was altered in the presence of human plasma, however changes in phenotype were strain dependent. For *S. aureus* MRSA3, the addition of plasma significantly enhanced biomass accumulation, whereas for *S. aureus* UAMS-1 the effect of plasma on biofilm formation seemed to be related to morphology, appearing more dense and compact compared to biofilms grown in only media (Figure 1A-B). Although coating of plates with 20% plasma v/v was previously reported to be optimal for promoting biofilm formation on glass coverslips, we observed that coating had a minimal effect on biofilm formation by the clinical isolates even at concentrations >5% v/v [19]. In contrast to these studies, and consistent with recent studies described in *Chen et al.*, supplementation of media with plasma between 10-25% v/v was observed to be a more optimal biofilm growth condition, significantly enhancing biofilm formation compared to biofilms grown under non-supplemented conditions or in wells coated with plasma at similar concentrations (Table 1) [18]. Notably, the effect of plasma on biofilm formation was isolate-dependent, with methicillin-resistant isolates appearing more responsive to plasma than the methicillin-susceptible isolate, UAMS-1 (Table 2).

**Table 1:** ***S. aureus *** **biofilm formation is more enhanced when plasma is part of the growth medium, compared to coating plates with plasma prior to biofilm formation**

**Strain**	**Percent plasma**	**Plasma coated**	**Plasma in medium**	**P-value**^**‡**^
		OD570 (mean + SD)*	**OD570 (mean + SD)**	
**MSSA UAMS-1**				
	0	0.61±0.34	0.87±0.31	
	1	0.81±0.41	1.63±0.48	0.15
	5	0.56±0.33	1.4±0.58	0.17
	10	0.25±0.08	2.4±0.46	0.01
	25	0.3±0.2	1.95±0.58	0.02
	50	0.32±0.17	0.83±0.76	0.22
**MRSA 1**				
	0	0.36±0.13	0.38±0.18	
	1	0.28±0.06	0.77±0.26	0.06
	5	0.67±0.47	1.08±0.49	0.22
	10	0.56±0.44	1.44±0.33	0.03
	25	0.58±0.27	1.22±0.3	0.08
	50	0.38±0.21	0.67±0.56	0.33
**MRSA 2**				
	0	0.38 + 0.13	0.6 ± 0.19	
	1	0.57 + 0.02	2.26 + 0.17	0.04
	5	0.79 + 0.47	2.37 + 0.1	0.04
	10	0.7 + 0.58	2.82 + 0.04	0.03
	25	0.67 + 0.49	2.47 + 0.6	0.04
	50	0.5 + 0.25	1.3 + 0.99	0.24
**MRSA 3**				
	0	0.58 + 0.31	0.53 ± 0.14	
	1	0.5 + 0.07	2.46 + 0.57	0.04
	5	0.93 + 0.69	2.26 + 0.71	0.11
	10	0.63 + 0.16	2.18 + 0.68	0.04
	25	0.53 + 0.51	2.5 + 0.37	0.02
	50	0.36 + 0.16	1.84 + 1.0	0.14

*96-well plates were coated with plasma for 24 hours, washed then biofilm was formed.

^‡^Plasma coated compared to plasma in the growth medium. P-values generated via One-way ANOVA with a Dunnett’s post hoc test.

**Table 2:** Increase in biofilm biomass measured by Crystal Violet method following incubation in plasma

	**UAMS-1**		**MRSA-1**		**MRSA-2**		**MRSA-3**	
	**OD**_**570**_**±SD**	**%±SD**	**OD**_**570**_**±SD**	**%±SDs**	**OD**_**570**_**±SD**	**%±SD**	**OD**_**570**_**±SD**	**%±SD**
**0%**	0.87±0.31	N/A	0.38±0.18	N/A	0.6 ± 0.19	N/A	0.53 ± 0.14	N/A
**1%**	1.63±0.48	87±29	0.77±0.26	102±34	2.26 ± 0.17*	277 ± 8ϯ	2.46 ± 0.57*	364 ± 23ϯ
**5%**	1.40±0.58	61±41	1.08±0.49*	189±45ϯ	2.37 ± 0.1*	295 ± 4ϯ	2.26 ± 0.71*	326 ± 31ϯ
**10%**	2.40±0.46	171±19	1.44±0.33*	279±23ϯ	2.82 ± 0.04*	370 ± 1ϯ	2.18 ± 0.68*	311 ± 31ϯ
**25%**	1.95±0.58	124±30	1.22±0.30*	221±25ϯ	2.47 ± 0.60*	317 ± 24ϯ	2.50 ± 0.37*	368 ± 15ϯ
**50%**	0.83±0.76	-5±92	0.67±0.56	76±84	1.30 ± 0.99	117 ± 76	1.84 ± 1.0	249 ± 54ϯ

*P ≤ 0.05 vs. 0% plasma, One-way ANOVA with Dunnet’s post hoc test.

ϯP ≤ 0.05 Percent increase in biofilm biomass (UAMS-1 vs. MRSA-1, MRSA-2, or MRSA-3) at comparable plasma percentages, One-way ANOVA with Dunnet’s post hoc test.

Results are expressed as Mean OD570 ± standard deviation and percent change from 0% plasma ± SD.

The observed variability of biofilm formation in response to plasma among the *S. aureus* isolates is not surprising, and likely indicates differences in genetic backgrounds and consequently in the binding affinity to plasma components, such as fibrinogen and fibronectin [20-22]. Indeed, as the coating of surfaces with plasma is thought to provide surface attachments, the differences in gene repertoire as well as expression can in part explain these observed differences. Importantly, the observation that supplementing media with plasma, rather than coating the surface, had a greater effect on biofilm formation suggests that the components of plasma may not only serve to facilitate attachment as previously thought, but may also impart changes to bacteria making them better suited for biofilm growth. In support of this, SEM analysis of biofilms grown with media supplemented with plasma demonstrated significant changes in the cell morphology of individual bacterial cells within biofilms, appearing as coccobacillus-like with a thicker, more heterogeneous appearing cell wall (Figure 1C-D). These changes to morphology, and likely to other properties of the biofilm, highlight the multiple effects that plasma can have in addition to the observed increases in biomass.

Although the effects of plasma have been previously reported, it is not completely understood how plasma enhances staphylococcal biofilm formation [7,18,19]. Staphylococci possess a number of surface expressed adhesins known as MSCRAMMs that facilitate attachment to host matrix molecules and are important for biofilm formation [5,6]. As these ligands are responsible for mediating attachment to surfaces, through interactions with host matrix components, we evaluated the effect of plasma on the expression of seven MSCRAMMs, including laminin binding protein (*eno*), encoding elastin binding protein (*ebps*), fibrinogen binding protein (*fib*), clumping factor A/B (*clfa/clfb*), and fibronectin binding protein A/B (*fbnA/fbnB*) following exposure to plasma. In response to plasma, significant changes in MSCRAMM gene expression were observed for all clinical isolates tested (Table 3). The effect of plasma on MSCRAMM gene expression was both isolate- and time-dependent. Of the genes evaluated, expression of the fibrinogen and fibronectin binding protein genes were most responsive to plasma exposure (Table 3).

**Table 3:** 10% Plasma alters MSCRAMM gene expression in a strain dependent fashion

**Strain**	**Gene**	Fold increase in gene expression*			
		**30 min**	**60 min**	**90 min**	**120 min**
**UAMS-1**	*Laminin binding protein (Eno)*	1.62	2.06	2.76	3.39
	*Encoding elastin binding protein (ebps)*	**3.42**	2.98	**4.61**	**3.50**
	*Fibrinogen binding protein (fib)*	0.00	**17.12**	**32.52**	**30.86**
	*Clumping factor A (clfA)*	2.08	**3.57**	**6.03**	**4.43**
	*Clumping factor B (clfB)*	1.04	**5.48**	2.33	**4.39**
	*Fibronectin binding protein (fbnA)*	**11.22**	**4.28**	**5.25**	1.96
	*Fibronectin binding protein (fbnB)*	**5.53**	**4.71**	**4.76**	**3.02**
**MRSA-1**	*Laminin binding protein (Eno)*	1.51	2.15	2.85	2.51
	*Encoding elastin binding protein (ebps)*	1.11	1.29	2.02	1.75
	*Fibrinogen binding protein (fib)*	**5.18**	**7.88**	**13.92**	**7.86**
	*Clumping factor A (clfA)*	2.88	2.82	3.69	4.10
	*Clumping factor B (clfB)*	0.98	1.62	2.73	2.32
	*Fibronectin binding protein (fbnA)*	**32.23**	**46.84**	**30.30**	2.79
	*Fibronectin binding protein (fbnB)*	0.87	**13.97**	**5.14**	**5.36**
**MRSA-2**	*Laminin binding protein (Eno)*	1.06	**3.53**	**3.83**	**3.63**
	*Encoding elastin binding protein (ebps)*	1.01	**3.80**	2.12	1.38
	*Fibrinogen binding protein (fib)*	1.30	**4.16**	**6.21**	**3.14**
	*Clumping factor A (clfA)*	**4.08**	**3.13**	**3.90**	2.62
	*Clumping factor B (clfB)*	1.14	1.60	2.50	**3.91**
	*Fibronectin binding protein (fbnA)*	2.59	**7.14**	**4.89**	2.00
	*Fibronectin binding protein (fbnB)*	**3.62**	**3.72**	**3.31**	2.61
**MRSA-3**	*Laminin binding protein (Eno)*	1.31	1.86	**3.96**	2.95
	*Encoding elastin binding protein (ebps)*	0.99	1.21	**3.67**	1.88
	*Fibrinogen binding protein (fib)*	2.44	**6.92**	**5.18**	**3.49**
	*Clumping factor A (clfA)*	1.82	1.73	**5.56**	2.73
	*Clumping factor B (clfB)*	1.32	1.15	**3.78**	**3.22**
	*Fibronectin binding protein (fbnA)*	2.57	2.08	**4.26**	**5.30**
	*Fibronectin binding protein (fbnB)*	2.60	2.35	**3.53**	**3.75**

*A greater than or equal to 3 fold increase was statistically significant (p<0.05) compared to baseline gene expression (t=0), and are indicated in boldface, One-way ANOVA with a Dunnet’s post hoc test.

The transition from planktonic to surface-attached growth results in significant changes in gene expression that may promote the biofilm mode of growth. Consistent with our observations, gene expression of MSCRAMMs during biofilm growth, including *fnbA/B*, *clfa*, *ebps*, and *fib*, have also been shown to be significantly enhanced during biofilm growth [23]. However, in these studies the expression of MSCRAMMs was optimally enhanced between 12 and 24 hours of biofilm growth. In contrast, we observed significant expression of the various MSCRAMM genes (>3 fold) after only 30 minutes following plasma exposure. This supports previous findings by *Chen* et al. demonstrating significant increases in biofilm biomass as early as six hours of growth compared to biofilms grown under normal conditions [18] and indicating that plasma enhanced biofilm formation may in part result from changes in gene expression of MSCRAMMs.

### Biofilms grown in the presence of plasma demonstrate increased tolerance to vancomycin *in vitro*

**Figure 2:**
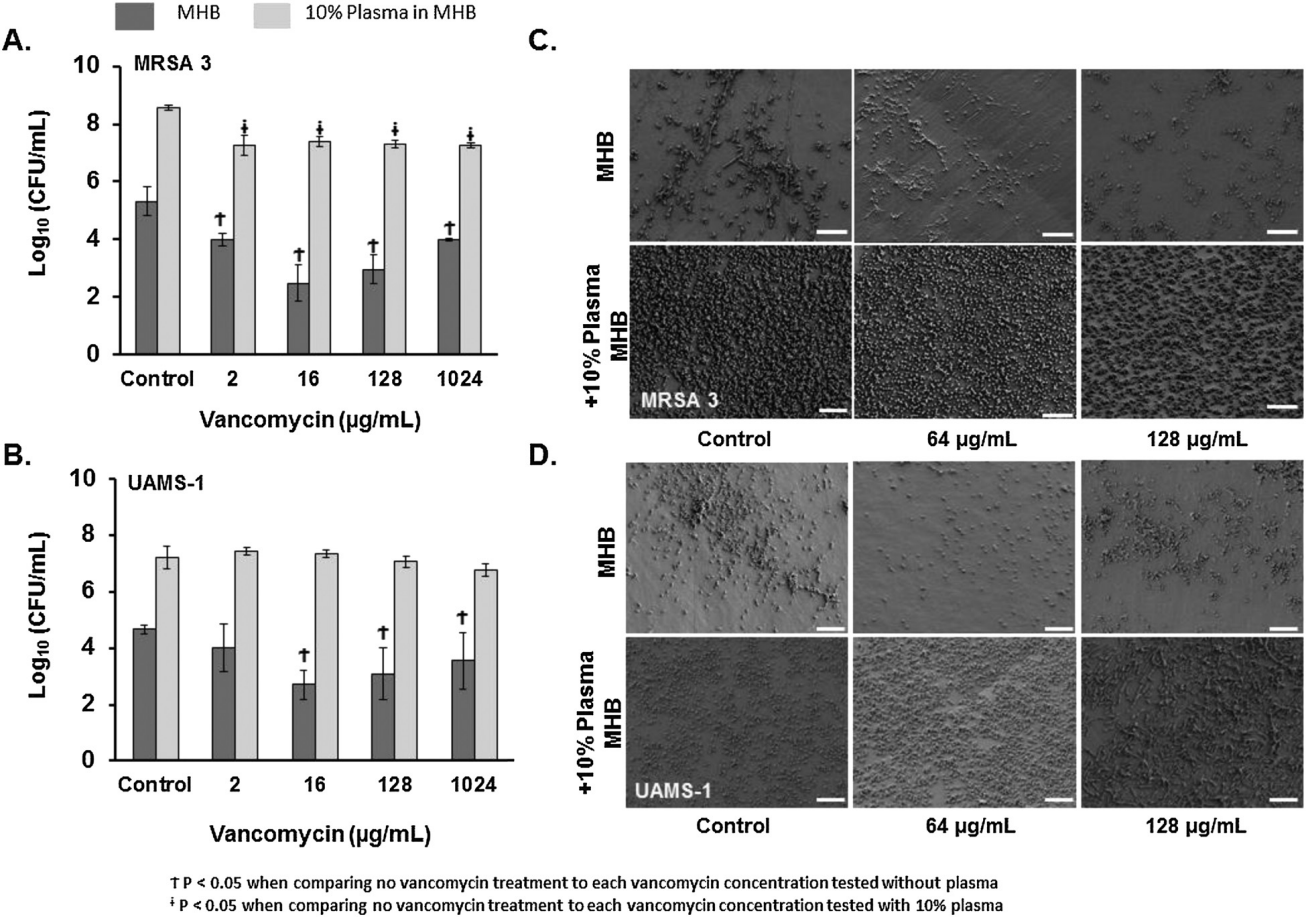
**Plasma augmented biofilms have reduced susceptibility to vancomycin *in vitro*. A-B)** Viability of bacteria (Log_10_ CFU/mL) within plasma augmented (MHB-10% Plasma, light bars) and non-augmented (MHB, dark bars) biofilms of a methicillin resistant (MRSA 3) and methicillin susceptible (UAMS-1) strain of *S. aureus* following overnight exposure to vancomycin (0, 2, 16, 128, and 1024 μg/mL). Bar graphs are indicative of the mean bacterial CFU ± SD. P-values generated via One-way ANOVA with a Dunnett’s post hoc test. **C-D)** SEM analysis of plasma and non-plasma augmented biofilms from the isolates listed above, following exposure to vancomycin. Images were captured at 2,500X. Size bars represent 10 μm.

Given the findings demonstrating the effects of plasma on bacterial gene expression and morphology, we next evaluated whether plasma augmentation of biofilms also had an effect on the susceptibilities of biofilms to antimicrobials using a well-described biofilm susceptibility assay [24]. In contrast to biofilms grown without plasma, biofilms grown in the presence of plasma demonstrated a significantly reduced susceptibility to vancomycin *in vitro* (Figure 2). Consistent with this, plasma augmented biofilms of a clinical methicillin resistant (MRSA3) and methicillin susceptible *S. aureus* (UAMS-1) isolate had greater numbers of viable bacteria, as determined by colony enumeration, following overnight exposure to vancomycin even at the highest concentrations tested compared to untreated controls (Figure 2A-B). In agreement with these results, SEM analysis of biofilms grown in the presence of plasma following exposure to vancomycin at similar concentrations demonstrated no visual reduction in attached, viable bacteria (Figure 2C-D).

One possible explanation for the observed increased resistance to vancomycin, could involve selection for persister cells [25]. Recent data in *Staphylococcus epidermidis* suggests that the number of persister cells, bacteria within the biofilm having low metabolic activity and accounting for the population resistant to antimicrobials, increases at high bacterial densities [26]. Although we did not assess for the presence of persister cells, plasma-augmentation was observed to increase bacterial density and by extension may have increased the number of persister cells within the biofilm. Alternately, the inoculum effect may also explain why plasma-augmented biofilms displayed reduced vancomycin susceptibility, as plasma-augmentation resulted in 3 log-units higher inoculum in the biofilms. Interestingly, vancomycin resistance in *S. aureus* has also been associated with increased cell wall thickness [27]. Given our observation by SEM that plasma treatment altered the *S. aureus* cell surface morphology (Figure 1, C-D), cell wall alteration may also have contributed to the overall reduced vancomycin susceptibility noted in this study. Nonetheless, the finding of reduced vancomycin susceptibility in plasma-exposed biofilms has important implications for evaluating activity of antimicrobials and novel treatments against biofilms *in vitro*. Based on these studies, the inclusion of host factors may be necessary to fully evaluate susceptibility of biofilm-associated organisms under conditions that simulate the *in vivo* environment.

## Conclusion

Most *in vitro* models used to study biofilms utilize incubation periods ranging from 12 to 48 hours, varying concentrations of ambient oxygen and carbon dioxide during incubation, varying incubation temperatures, and often omit potentially important host factors [8,28,29]. Consequently, although useful for high throughput analysis, *in vitro* models may not represent the true capacity of pathogens to form biofilms *in vivo*[8,28,29]. Thus there is a need for standardization in biofilm testing methods which is currently lacking [8].

While plasma has been previously demonstrated to increase biofilm formation [5-9,18,19], our study has extended this observation by examining the impact of plasma on several clinical isolates, demonstrating that host factors, including human plasma, can influence cell morphology and *S. aureus* gene expression, which may favor the biofilm phenotype.

These findings indicate a role for inclusion of host components into models evaluating biofilm formation as it would be more representative of *in vivo* conditions and may more accurately facilitate clinically relevant biofilm studies.

## Methods

### Bacterial isolates and growth conditions

Three clinical methicillin-resistant *S. aureus* (MRSA) isolates collected during clinical care from inpatients at our facility, previously demonstrated to be strong biofilm-formers, were selected for study [30]. UAMS-1 (ATCC 49230), is a well-characterized, methicillin-susceptible osteomyelitis isolate of *S. aureus*[31]. The other isolates in this study were isolated from the following anatomic sites: bone (UAMS-1), nares (MRSA-1, MRSA-3), and the trachea (MRSA-2). The isolates also represent a variety of pulsed-field gel electrophoresis types: USA100 (MRSA-1), USA200 (UAMS-1), USA300 (MRSA-3), and USA800 (MRSA-2). Bacterial cultures were maintained at -80°C and sub-cultured on sheep’s blood agar plates (Remel, Lenexa, KS, USA) overnight at 37°C prior to each experimental assay. Bacteria were grown in Cation-Adjusted Mueller-Hinton Broth (MHB) (Becton Dickinson, Franklin Lakes, NJ). UAMS-1 and MRSA-3 were selected for CLSM, SEM, and antimicrobial susceptibility testing.

### Antimicrobial agents and reagents

Human plasma was purchased from the Biological Specialty Corporation (Colmar, PA). Plasma was filtered using 100 μm cell isolators (BD Falcon, Franklin Lakes, NJ) and diluted in MHB at 1, 5, 10, 25, and 50% (v/v). Vancomycin powder was purchased from Sigma-Aldrich (St Louis, MO).

### Biofilm formation in 96-well microtiter plates

Biofilm formation was assessed by measuring the accumulation of biomass in sterile 96-well flat-bottom polystyrene plates (Costar, Corning Incorporated, Lowell MA) as previously described following 24 h incubation [30]. Briefly, 10 μL of overnight bacterial suspensions (~10^8^ CFU) were added to wells containing 190 μL media supplemented with or wells coated with various concentrations of human plasma. Following 24 h incubation at 37°C, wells were washed with PBS, stained with 0.1% crystal violet, and biomass was quantified by measuring the optical density at 570 nm (*OD*_570_) of the supernatant following solubilization in ethanol. All experiments were performed in triplicate.

### Confocal laser scanning microscopy (CLSM)

Biofilm formation by select clinical isolates was carried out on glass chamber slides (Thermo Scientific-Nunc, Rochester, NY) following 24 h exposure to media with or without 10% plasma by CLSM as previously described [19]. Briefly, following biofilm growth cells were washed with PBS and fixed with 4% formaldehyde. Biofilms were stained with a biofilm cell stain to visualize the extracellular polymeric matrix and bacterial cells according to the manufacturer’s instructions (Molecular Probes, Eugene, OR). CLSM images were acquired using an Olympus FluoView 1000 Laser Scanning Confocal Microscope (Olympus America Inc., Melville, NY) under 20X magnifications using an argon laser at 488 nm and a HeNe-G laser at 543 nm. Image analysis/processing were via Olympus FluoView software. Images were acquired from at least three distinct regions on the slide and representative images were selected.

### Scanning electron microscopy (SEM)

Biofilm formation was carried out for 24 h as described above in the presence or absence of 10% plasma, using the MBEC™ P&G from Innovotech (Edmonton, Alberta, Canada). Following incubation, individual pegs were removed and fixed with 2% (w/v) gluteraldehyde, 2% (w/v) paraformaldehyde, 0.15 M sodium cacodylate, 0.15% (w/v) alcian blue. Pegs were rinsed thrice with 0.15 M sodium cacodylate buffer, immersed in 1% (v/v) osmium tetroxide in sodium cacodylate buffer and incubated for 1 hr at room temperature. Pegs were rinsed thrice with distilled water followed by dehydration with an ascending series of ethanol (50%, 75%, 95%, and 100%). Samples were treated with hexamethyldisilizane for 5 minutes, re-submerged and allowed to evaporate at room temperature prior to mounting. Copper tape was used to secure the pegs to carbon tape on stubs to reduce charging artifact. Samples were sputter coated with gold and viewed with a Zeiss Sigma VP scanning transmission electron microscope.

### RNA isolation and quantitative real time reverse transcription PCR (qRT-PCR)

Bacteria were grown to an OD_600_ of 0.2 (~10^8^ bacteria/ml), washed with 1X PBS, and added to MHB media supplemented with 10% human plasma at a final concentration of 2 x 10^8^ CFU/mL. Bacteria were incubated in MHB with 10% (v/v) plasma for 0, 30, 60, 90, and 120 min at 37°C. Time points were chosen based on previous studies determined to be optimal for evaluating expression of MSCRAMMs following exposure to host components [32]. In addition, these time-points were chosen as the exopolysaccharide of the developing biofilm is visible in just 5 hours after inoculation and has the characteristics of a mature biofilm by 10 hours have been, and if plasma were to enhance biofilm formation via increased MSCRAMM expression, gene expression favoring biofilm formation would be induced in planktonic bacteria soon after exposure, prior to biofilm formation and maturation [33].

At the indicated timepoints, bacteria were harvested by centrifugation, washed, and RNA was isolated using an RNeasy Mini Kit (Qiagen, Valencia, CA) combined with Bacterial-RNA Protect (Qiagen) following the manufacturer’s instructions. Bacteria were lysed by treating cells with lysostaphin (200 μg/ml) (Sigma-Aldrich, St. Louis, MO) for 30 min at 37°C (30). RNA was isolated, and reverse transcribed using the High Capacity cDNA reverse transcription kit (Life Technologies, Grand Island, NY), per the manufacturers’ protocol. Quantitation of gene expression was via TaqMan (Life Technologies) methodology using the relative standard curve method. The gene specific PCR primers were developed with Primer Express software (Life Technologies) (Table 1). Gyrase B expression was the endogenous control. Real-time quantitative PCR reactions were performed with 1.5 ng of total RNA converted to cDNA template. PCR reactions consisted of the cDNA template, 1X Universal PCR Master Mix for Gene Expression (Life Technologies), gene specific primers (900 nM) and probe (250 nM) in a total volume of 20 μL. Standard curves consisted of ten-fold dilutions of a positive control sample. PCR reactions were performed in triplicate and cycled in a 7900HT Sequence Detection System using standard protocols (Applied Biosystems, Grand Island, NY). Transcript levels were normalized to the internal control mRNA and fold-regulation changes was calculated using 2^–ΔΔCt^ method. Fold-gene expression equal or greater than 3-fold was determined to be statistically significant (*p < 0.05*), following analysis using a 1-way ANOVA with a Dunnett’s post hoc test comparing treatment groups to the control group.

### Viability of biofilm-derived bacteria after vancomycin exposure

Biofilm formation was carried out as described above using the MBEC™ P&G. Briefly, bacteria were inoculated into wells containing either MHB with or without plasma (10%), covered with a lid containing pegs for the attachment of the bacteria, and incubated at 37°C for 24 hr with agitation. Following incubation, plate lids containing the pegs with the attached biofilms were washed with PBS, placed into 96 well plates containing 2-fold serial dilutions of antibiotics diluted in MHB, and incubated overnight at 37°C. To determine the viability of biofilm-derived bacteria following vancomycin exposure, MBEC plate lids were washed, placed into 96-well plates containing MHB, and biofilm-derived bacteria were detached from the pegs of lids by sonication for 5 min. Plate lids with pegs were removed and replaced with a conventional microtiter plate lid. Bacterial viability was determined by plating serial dilutions on blood agar plates at 0, and 6 hours post-exposure as per a previous study [34].

### Statistical analysis

All experiments were performed in triplicate with a minimum of three technical replicates per individual experiment. The data sets were normally distributed, and for all quantitative studies a One-way ANOVA with a Dunnett’s post hoc test was used to compare all treatment groups to the control group. P-values of ≤0.05 were considered to be statistically significant. Statistical software utilized was SigmaStat® Version 12.0.

## Competing interests

The authors declare that they have neither financial nor non-financial competing interests.

## Authors’ contributions

APC, CJS, KSA and JCW conceived the study and participated in its design. APC, CJS, DRR, MES, and SKH conducted all experiments and collected data. APC, CKM, KSA, and JCW helped to draft the manuscript. APC and CJS performed the statistical analysis. All authors read and approved the final manuscript.
